# Intravenous Lormetazepam during Sedation Weaning in a 26-Year-Old Critically Ill Woman

**DOI:** 10.1155/2014/372740

**Published:** 2014-10-08

**Authors:** Alawi Luetz, Bjoern Weiss, Claudia D. Spies

**Affiliations:** Department of Anesthesiology and Intensive Care Medicine, Charité-Universitätsmedizin Berlin, Campus Charité Mitte and Campus Virchow-Klinikum, Augustenburger Platz 1, 13353 Berlin, Germany

## Abstract

Recent evidence revealed that sedation is related to adverse outcomes including a higher mortality. Despite this fact, patients sometimes require deep sedation for a limited period of time to control, for example, intracranial hypertension. In particular in these cases, weaning from sedation is often challenging due to emerging agitation, stress, and delirium. The submitted research letter reports a rare case of severe and persisting agitation that was unresponsive to all available treatments. Ultimately, lormetazepam which has recently become available for intravenous use in Germany resolved the problem by stress-reduction and anxiolysis without leading to measurable sedation.

## 1. Introduction

Sedation in critically ill patients is associated with an increased risk of death within 6 months [1, 2]. Administering drugs with stress-reducing and anxiolytic effects is often necessary to ensure that the patient is calm, alert, and attentive. Despite this fact, patients sometimes require deep sedation for a limited period of time to control, for example, intracranial hypertension or tolerate prone positioning. In particular in these cases, weaning from sedation is often challenging due to agitation, stress, and additional symptoms that may be associated with delirium.

## 2. Case Presentation

The 26-year-old, female, patient was admitted to our intensive care unit (ICU) from an external hospital, suffering from severe acute respiratory distress syndrome due to community-acquired pneumonia. At the time of admission, she was mechanically ventilated and deeply sedated (Richmond Agitation Sedation Scale (RASS) −5) with continuous infusion of midazolam and propofol. Analgesia was performed with continuous infusion of sufentanil. Beside starting an empiric antimicrobial therapy, we used prone positioning to stabilise oxygenation. After clinical situation regarding respiration had improved, we initiated daily spontaneous awakening trials (SATs). However, during SATs, the patient was severely agitated (RASS +3) and delirious (confusion assessment method for the ICU (CAM-ICU) positive) and required immediate deep sedation due to respiratory deterioration.

During the SATs the Behavioural Pain Scale was always <6. We initiated a symptom-oriented therapy for delirium with haloperidol and clonidine without remarkable success.

A computerised tomography scan (day 5) showed an infarction of the right cerebellar hemisphere and signs of increased intracranial pressure which made a constant deep sedation necessary. Furthermore, the patient suffered from a pulmonary reinfection with a consecutive CO_2_-retention. Therefore, an extracorporeal lung-assist (pECLA) was necessary to optain normocapnia and control intracranial pressure consecutively. After improvement of the clinical situation, we restarted daily SATs. Following, we faced the same problem of severe agitation. Symptom-oriented treatment of delirium was continued with haloperidol and alpha-2-agonists (clonidine followed by dexmedetomidine).

A cranial magnetic resonance imaging showed residues of very small cortical and subcortical bleedings which were classified to be of septic-embolic pathogenesis. However, in accordance with our neurosurgeons and neurologists, these findings could not explain agitated delirium during the SATs. A lumbar puncture revealed no pathological findings.

In order to stop propofol infusion and to reduce admnistration of midazolam, we perfomed inhalative sedation with isoflurane (day 27). Although we were able to stop propofol and midazolam infusion, we were not able to achieve a RASS between −2 and +1.

**Figure 1:**
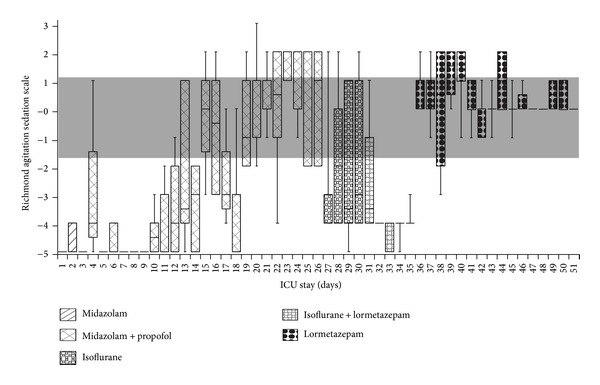
Levels of sedation and agitation. Measurement with the Richmond Agitation Sedation Scale (RASS) during intensive care unit treatment. The shadow indicates RASS −1 to 0 (no sedation). Analgesia was performed with continious infusion of sufentanil until discharge from our intensive care unit.

**Figure 2:**
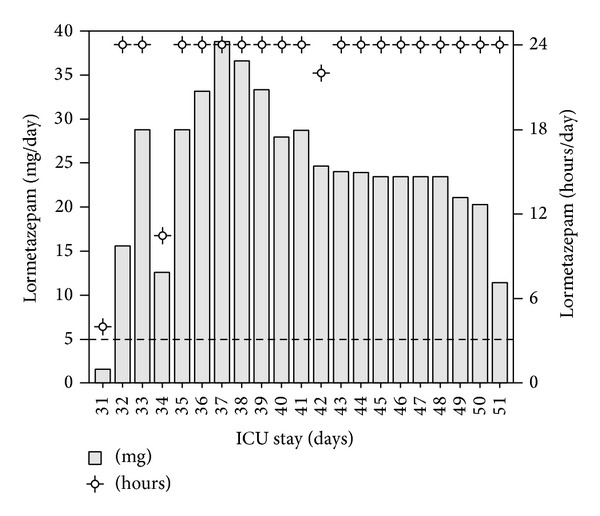
Duration and amount of intravenous administered lormetazepam per day. mg = milligram.

On day 31 after ICU admission, we started intravenous administration of lormetazepam which is newly available for iv use in Germany. The initial infusion rate was 0.36 *μ*g/kg/hr. Even though the patient was less agitated during SATs, we still measured RASS peaks of >1. Consequently, we increased the amount of continuously administered lormetazepam which lead to a constant improvement. On day 36, the patient had a RASS of 0 for several hours (Figure 1). At this time, the patient received continuous lormetazepam infusion with an unexpected high dose of 33 mg/day (Figure 2), dexmedetomidine infusion of 1.4 *μ*h/kg/hr, and 3 × 2 mg haloperidol intravenously. Even though the patient was still CAM-ICU positive but not agitated, we started weaning the patient from the ventilator. Furthermore, we reduced administration of dexmedetomidine and sufentanil about 10% per day. In order to maintain a RASS of 0 to −1, lormetazepam infusion had to be increased to a maximum dose of 39 mg/day (Figure 2). Because of the persistent positive CAM-ICU, we decided to use quetiapine instead of haloperidol for antipsychotic therapy. Within the next 2 weeks, the patient was successfully weaned from the respirator. With ICU discharge (day 51), sufentanil and dexmedetomidine infusion were stopped. After 3 consecutive days without delirium, antipsychotic therapy was stopped as well. Two milligrams of lormetazepam was administered orally every 8 hours.

**Figure 3:**
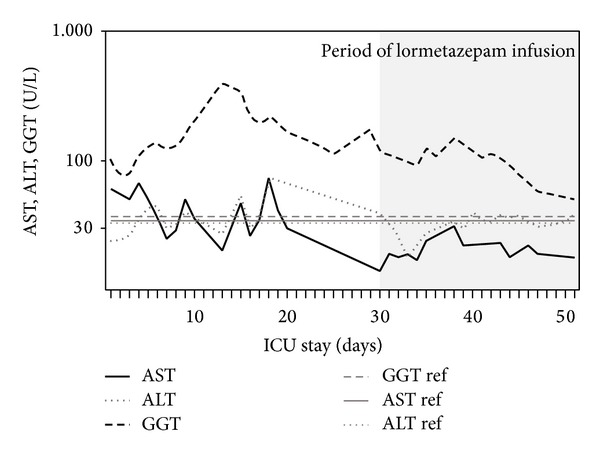
Blood concentrations of different liver enzymes during ICU treatment. (AST, aspartate transaminase; ALT, alanine transaminase; GGT, gamma-glutamyl transpeptidase). The grey lines (GGT ref, AST ref, and ALT ref) indicate the upper threshold levels for the corresponding enzymes; ref = reference.

No obvious relationship between an increase of liver enzymes and the administration of lormetazepam was determined. In fact, blood concentrations of the alanine transaminase (ALT) and the gamma-glutamyl transpeptidase (GGT) tended to decrease with the start of lormetazepam infusion (Figure 3). Blood concentrations of the aspartate transaminase (AST), creatinine, and urea were normal during all times of lormetazepam infusion.

## 3. Discussion

Since 2009, intravenous lormetazepam is approved for anxiolysis and sedation in critically ill patients in Germany. Unlike many other hypnotics, the distribution of lormetazepam is associated with less alteration of rapid eye movement sleep [3]. Furthermore, lormetazepam showed superior anxiolytic properties compared to other benzodiazepines when used for premedication [4]. Lormetazepam is metabolised independently from cytochrome-P450 enzymes and inactivated by glucoronidation. The resulting inactive metabolites are renally excreted afterwards. The elimination half-life of lormetazepam after intravenous administration is therefore constantly 8–12 hours and shorter than the half-life of lorazepam.

In our case, lormetazepam was able to sufficiently treat agitation without sedating the patient (RASS > −2). Clinically, it seemed that lormetazepam caused predominantly anxiolytic effects accompanied by an improved navigability of the drug.

From the pharmacodynamic point of view, benzodiazepines act by indirect activation of the GABA_A_ receptor that either enhance or reduce the inhibitory effects of GABA [5]. The majority of brain GABA_A_ receptors contain *α*-, *β*-, and *γ*-subunits. Studies could show that variations in the *α*-subunit are the main determinants of benzodiazepines pharmacology. Löw and colleagues could show that the anxiolytic effect of benzodiazepines is mediated by *α*_2_-GABA_A_ receptors but not by *α*_3_-GABA_A_ receptors [6]. Because nonselective benzodiazepines, exemplified by diazepam, act by enhancing the inhibitory effects of GABA at (A) receptors containing either an *α*_1_, *α*_2_, *α*_3_, or *α*_5_ subunit, such compounds possess a relatively narrow window between doses that produce anxiolysis and those that cause sedation. That is probably why, in the clinical setting, the proven anxiolytic efficacy of benzodiazepines is often superimposed by their sedative effect. A hypothesis for the different clinical effect of lormetazepam compared to lorazepam and midazolam might be that lormetazepam exerts its effect by *α*_2_-GABA_A_-receptor-agonistic activity, which is likely to be responsible for the antianxiety-like effects of benzodiazepines.

Although we needed remarkable higher daily doses as recommended by the manufacturer, continuous, intravenous administration of lormetazepam was safe, without any observed side effects, related to the drug.

Randomized controlled trials are needed to investigate whether administration of lormetazepam compared to othere benzodiazepines can be benificial for the ICU patient.
